# The Roles of Exosome-Derived microRNAs in Cardiac Fibrosis

**DOI:** 10.3390/molecules29061199

**Published:** 2024-03-07

**Authors:** Xinyuan Tang, Mingyang Leng, Wenyue Tang, Zhenlu Cai, Lin Yang, Liang Wang, Yue Zhang, Jiao Guo

**Affiliations:** 1 Guangdong Metabolic Diseases Research Center of Integrated Chinese and Western Medicine, Guangzhou 510006, China; tangxinyuan9917@outlook.com (X.T.); lengmingyang123@outlook.com (M.L.); norahtang97@outlook.com (W.T.); c17370734554@outlook.com (Z.C.); a1070298016@outlook.com (L.Y.); wl1026815@outlook.com (L.W.); 2 Key Laboratory of Glucolipid Metabolic Disorder, Ministry of Education of China, Guangzhou 510006, China; 3 Guangdong Key Laboratory of Metabolic Disease Prevention and Treatment of Traditional Chinese Medicine, Guangzhou 510006, China; 4 Institute of Chinese Medicine, Guangdong Pharmaceutical University, Guangzhou 510006, China

**Keywords:** exosomes, cardiac fibrosis, miRNA, cardiovascular diseases, extracellular vesicles

## Abstract

Cardiovascular disease (CVD) stands as the foremost cause of patient mortality, and the lack of early diagnosis and defined treatment targets significantly contributes to the suboptimal prevention and management of CVD. Myocardial fibrosis (MF) is not only a complex pathogenic process with no effective treatment currently available but also exerts detrimental effects on the progression of various cardiovascular diseases, thereby escalating their mortality rates. Exosomes are nanoscale biocommunication vehicles that facilitate intercellular communication by transporting bioactive substances, such as nucleic acids and proteins, from specific cell types. Numerous studies have firmly established that microRNAs (miRNAs), as non-coding RNAs, wield post-transcriptional regulatory mechanisms and exhibit close associations with various CVDs, including coronary heart disease (CHD), atrial fibrillation (AF), and heart failure (HF). MiRNAs hold significant promise in the diagnosis and treatment of cardiovascular diseases. In this review, we provide a concise introduction to the biological attributes of exosomes and exosomal miRNAs. We also explore the roles and mechanisms of distinct cell-derived exosomal miRNAs in the context of myocardial fibrosis. These findings underscore the pivotal role of exosomes in the diagnosis and treatment of cardiac fibrosis and emphasize their potential as biotherapies and drug delivery vectors for cardiac fibrosis treatment.

## 1. Introduction

According to the World Health Organization, it is estimated that, by 2030, cardiovascular disease (CVD) will claim the lives of approximately 25 million people [1]. CVD continues to stand as the foremost threat to human health [2]. Numerous studies have demonstrated that myocardial fibrosis, resulting from an imbalance in the synthesis and degradation of the extracellular matrix (ECM), an imbalance in the proportions of various collagen types, and disrupted arrangement, serves as an inevitable process leading to the terminal stages of various clinical cardiovascular diseases such as atrial fibrillation, arrhythmias, heart failure, and even sudden cardiac death. It serves as a primary manifestation of cardiac structural remodeling. Therefore, the identification of novel targets for the early diagnosis and prevention of myocardial fibrosis holds immense clinical significance.

Recent research has unveiled the involvement of exosomal intercellular communication in cardiac fibrosis [3,4]. Exosomes are a subtype of small extracellular vesicles (EVs) that originate from the endosomal system and range in size from 30 to 200 nm [5]. These exosomes serve as vital conduits for intercellular communication and can exert critical regulatory functions in cardiovascular diseases by transporting bioactive molecules, such as microRNAs (miRNAs) [6]. Although exosomes have been implicated in the diagnosis and treatment of various diseases in recent years, their role in myocardial fibrosis has remained unclear. This article aims to review recent advances in research regarding the interplay between exosomal miRNAs and cardiac fibrosis, explore potential mechanisms through which exosomal miRNAs affect myocardial fibrosis, and assess their value in the diagnosis and treatment of CVD.

## 2. Myocardial Fibrosis

CVD is a global public health concern characterized by increasing morbidity and mortality rates each year. Among the various CVDs, heart failure (HF) represents the end stage and final outcome, and myocardial fibrosis (MF) is closely associated with nearly all CVDs, particularly HF. Myocardial fibrosis represents an inevitable progression in heart conditions, including conditions such as hypertension, coronary heart disease, and cardiomyopathy, ultimately leading to HF. It can manifest in the early stages of heart injury and persist throughout the course of heart disease, making it a pivotal pathophysiological process in the development of HF.

Fibrosis serves as a crucial mechanism for tissue remodeling in response to various organ injuries and is a fundamental part of tissue repair. The accumulation of extracellular matrix (ECM) components, such as collagens and fibronectin, is a normal and essential process during the repair of minor or non-repetitive organ injuries. However, in cases of severe or repetitive injuries, damaged tissue fibroblasts become overactive, leading to an excessive buildup of ECM. This, in turn, results in pathological changes within the interstitial tissue, leading to scarring, organ dysfunction, and, in some cases, organ failure. Excessive fibrosis is a major contributor to several chronic diseases, including interstitial lung disease, progressive cirrhosis, systemic sclerosis, diabetic nephropathy, diabetic eye disease, and cardiovascular disease [7].

**Figure 1:**
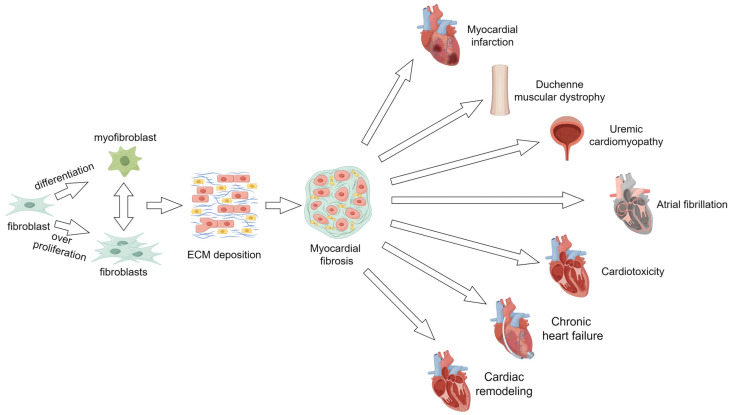
The relationship between myocardial fibrosis and various diseases. Excessive proliferation of cardiac fibroblasts or differentiation into myofibroblasts will cause increased extracellular matrix deposition. When the extracellular matrix environment is not balanced, myocardial fibrosis occurs, leading to various diseases.

Myocardial fibrosis, characterized by the excessive accumulation of interstitial extracellular matrix (ECM) and thickening of the basement membrane, represents a significant pathological feature in various heart diseases and is a key contributor to cardiac dysfunction. Clinical data reveal elevated ECM deposition and myocardial fibrosis in the interstitial heart tissues of patients suffering from advanced HF [8]. Consequently, it holds immense significance to explore the mechanisms underlying the development of myocardial fibrosis, diagnose and assess myocardial fibrosis at the earliest stages, and implement effective measures to delay or even prevent the progression of myocardial fibrosis. Multiple evidence suggests that alleviating myocardial fibrosis can improve survival rates and enhance the quality of life for patients dealing with various diseases (as illustrated in Figure 1).

### 2.1. Myocardial Fibrosis and Myocardial Infarction

Myocardial infarction (MI) continues to be a leading cause of morbidity and mortality on a global scale. Following a myocardial infarction, various pathological changes occur within the heart, with myocardial fibrosis playing a pivotal role in the regulation of cardiac function. It influences the development of heart failure and has a significant impact on the size, shape, and wall thickness of the ventricles [9,10,11]. Increasing evidence supports the notion that excessive fibrosis can lead to ventricular dilation, worsen the extent of infarction, and contribute to HF [12,13,14]. Consequently, the inhibition of myocardial fibrosis holds promise for potential clinical therapeutic effects [15,16,17].

### 2.2. Myocardial Fibrosis and Duchenne Muscular Dystrophy

The most common form of these inherited disorders is Duchenne muscular dystrophy (DMD) [18], a degenerative, X-linked disease affecting both skeletal and cardiac muscles, caused by mutations in the dystrophin gene. With the advancement of patient care and respiratory support technologies, myocardial fibrosis-induced cardiac hypertrophy imbalance has become the leading cause of death in DMD patients [19]. A comprehensive understanding of the occurrence, progression, and molecular regulatory mechanisms of myocardial fibrosis can aid in the identification of drug targets and the development of effective therapeutic approaches to improve cardiac function in DMD.

### 2.3. Myocardial Fibrosis and Uremic Cardiomyopathy

Uremic cardiomyopathy (UCM) is a complication of nephropathy, primarily characterized by metabolic remodeling, cardiac hypertrophy, myocardial fibrosis, and cardiac inflammation [20,21]. When cardiomyocytes are exposed to uremic serum, macrophage infiltration occurs, leading to the transfer of miR-155 [22] from macrophage-derived exosomes to cardiomyocytes. This transfer inhibits the translation of forkhead transcription factor class O (FoxO3a) in cardiomyocytes, ultimately promoting cardiomyocyte pyroptosis and ameliorating uremic cardiomyopathy, which includes heart hypertrophy and fibrosis.

### 2.4. Myocardial Fibrosis and Atrial Fibrillation

Atrial fibrillation (AF) significantly increases the mortality and morbidity associated with arrhythmias [23]. In recent years, mounting evidence supports the notion that inflammation, myocardial fibrosis, and cardiac remodeling may play crucial roles in AF. However, there is still no definitive mechanism to explain the onset and progression of this disease. AF is triggered by various factors, with cardiac remodeling serving as the pathophysiological basis, and myocardial fibrosis [24] being a significant manifestation of cardiac remodeling.

### 2.5. Myocardial Fibrosis and Cardiotoxicity of Antitumor Drugs

Numerous epidermal growth factors, including the receptor tyrosine protein kinase ErbB2/HER2, exhibit upregulation during cancer development [25]. Trastuzumab (Trz) was the pioneering monoclonal antibody designed to combat cancer by inhibiting the ErbB2 pathway [26]. ErbB2 is also expressed in cardiomyocytes, and when stimulated by neuregulin-1, a ligand from the receptor tyrosine kinase family, ErbB2 activates downstream signaling pathways such as ERK1/2-MAPK and PI3K-Akt, thereby promoting the survival of stressed cardiomyocytes [27]. The combined treatment of Trz and Doxorubicin (Dox, an anthracycline) has been shown to enhance treatment efficacy, albeit at the cost of increased cardiotoxicity [28]. Research data suggest that overexpression of ErbB2 results in the upregulation of antioxidant enzymes, reduces the levels of basal reactive oxygen species (ROS), and mitigates the cardiotoxic effects of Dox [29]. However, Trz inhibits ErbB2, potentially exacerbating cardiotoxicity [30]. Consequently, in clinical practice, Dox and Trz are typically administered separately to manage cardiotoxicity such as myocardial fibrosis [31].

### 2.6. Myocardial Fibrosis and Chronic Heart Failure

Chronic heart failure (CHF) ranks among the leading causes of high mortality attributed to heart disease [32]. The pathophysiology of CHF is intricate, characterized by left ventricular remodeling, which results in increased wall mass, chamber enlargement, and a more spherical chamber configuration. These changes collectively contribute to the progressive decline in left ventricular performance and play a pivotal role in the development of CHF [33,34]. Notably, among all the pathogenic processes involved in left ventricular remodeling, myocardial fibrosis stands out as a key determinant.

### 2.7. Myocardial Fibrosis and Cardiac Remodeling

Cardiac remodeling is a crucial factor in the progression of HF, and one of its indicators is the presence of cardiac fibrosis. Numerous studies have explored the potential of various miRNAs as diagnostic markers for heart failure and as tools to gauge the disease’s progression [35,36]. Furthermore, miRNAs encapsulated in serum exosomes have emerged as pivotal players in the context of CVDs [37].

## 3. The Biogenesis and Composition of Exosomes

In 1981, EG Trams [38] discovered small vesicles surrounded by a bilayer membrane in the supernatants of cultured sheep reticulocytes. These vesicles, later identified by Johnstone [39] in 1987 as exosomes, were initially thought to be cellular waste bins or discarded materials. Recent studies in the field of biological science have revealed that exosomes play crucial roles in a variety of biological and pathological processes.

### 3.1. Exosomal Biogenesis

**Figure 2:**
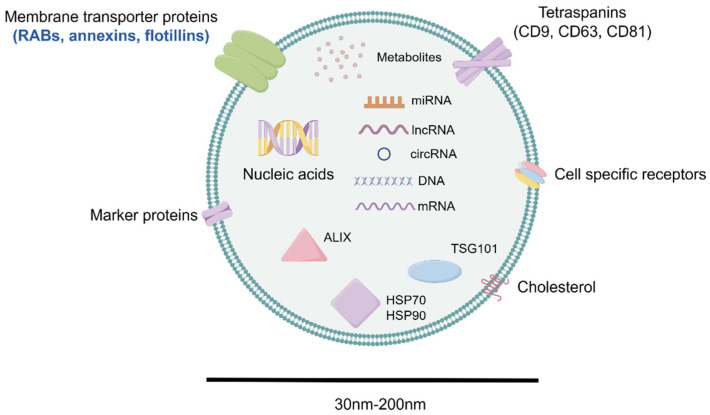
The composition of exosome. Exosomes ranging in size from 30 to 200 nm contain a large number of proteins, including membrane transport proteins such as RAB GTPases, annexins, flotillins, ALIX from multivesicular bodies (MVBs), and TSG101. In addition, exosomes host tetraspanin transmembrane proteins, such as CD9, CD63, and CD81, and heat shock proteins, including HSP60 and HSP90. Furthermore, exosomes encapsulate a variety of nucleic acids, such as miRNA, mRNA, lncRNA, tRNA, snRNA, snoRNA, and circRNA, and lipids.

The release of extracellular vesicles (EVs), which are particles separated by lipid bilayers and incapable of self-replication, is a universal phenomenon observed in all cells [5]. EVs can be roughly composed of ectosomes and exosomes [40]. Ectosomes are vesicles that form from outward budding of the plasma membrane surface, encompassing microvesicles, microparticles, and large vesicles ranging in size from approximately 50 nm to 1 μm in diameter. Exosomes (as illustrated in Figure 2) are small, membrane-bound vesicles and a unique class of EVs by virtue of their biogenesis, ranging in size from 30 to 200 nm and originating from multivesicular endosomes (MVEs), also known as multivesicular bodies (MVBs). The cell membrane initially internalizes to generate endosomes, from which a portion of the endosomal membrane invaginates, forming small vesicles and eventually leading to the formation of MVBs. Ultimately, MVBs fuse with the membrane, releasing the intraluminal endosomal vesicles into the extracellular space where they become exosomes [41].

### 3.2. Exosomal Composition

Exosomes serve as vehicles for transferring macromolecules derived from various tissues and organs, encompassing a wide array of proteins, nucleic acids, and lipids. These components predominantly facilitate cellular communication between different cell populations within multicellular organs, contributing to the maintenance of cellular homeostasis. Exosomes typically contain a repertoire of proteins, including membrane transport proteins such as RAB GTPases, annexins, flotillins, ALIX from multivesicular bodies (MVBs), and TSG101. Additionally, exosomes house tetraspanin transmembrane proteins, such as CD9, CD63, and CD81, and heat shock proteins, including HSP60 and HSP90. Furthermore, exosomes encapsulate various nucleic acids, such as miRNA, mRNA, lncRNA, tRNA, snRNA, snoRNA, and circRNA, which act as regulators of gene expression and hold potential as biomarkers [42,43,44,45].

Although it was initially considered that the primary function of exosomes was to eliminate cellular waste, with the increasing understanding of exosomal structural and physiological functions, an important focus in exploring the pathobiology of disease is to dig into cell-specific exosomes. Based on the wide range of contents packaged in exosomes, the regulation of exosome contents is a potential therapeutic strategy for the treatment of heart disease.

## 4. Exosomal miRNA

MiRNA, first discovered in 1993, is an endogenous non-coding RNA consisting of 19–25 nucleotides [46,47]. MiRNAs primarily bind to the 3′ untranslated region (3′UTR) of target gene mRNA, thereby regulating the expression of these target genes and subsequently influencing various physiological processes [48].

### 4.1. Exosomal miRNA Transport

**Figure 3:**
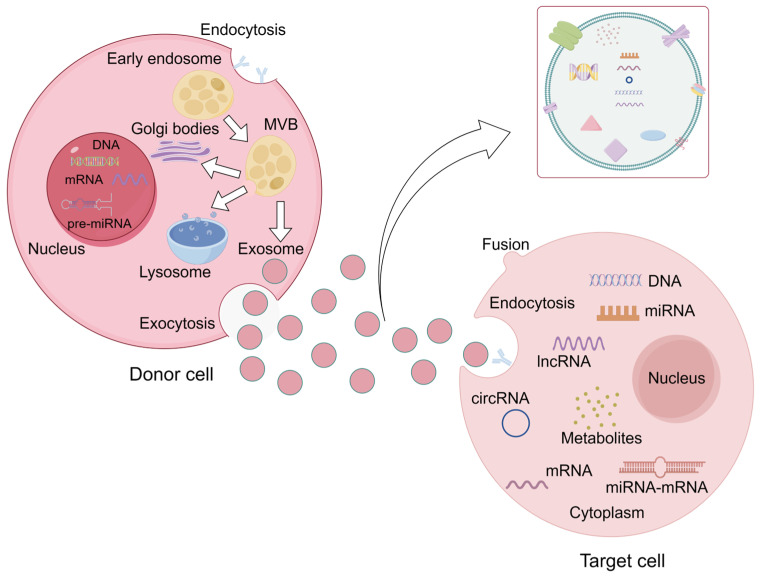
The secretion of exosomal miRNA. Firstly, miRNAs are selectively encased in MVBs, and then MVBs fuse with the donor cell membrane to release exosomes into the extracellular environment. Secondly, the target cells internalize the exosomes attached to the cell membrane through endocytosis, and finally release their contents into the cell. The released miRNA specifically binds to the 3′UTR of the target mRNA and inhibits the expression of the target gene.

MiRNA constitutes a significant portion of the contents within exosomes, and the phospholipid bilayer of exosomes safeguards miRNA from degradation. When exposed to different cellular microenvironments, cells actively and selectively encapsulate miRNAs, releasing them into the extracellular environment within exosomes in response to various stimuli. Stoorvogel et al. [49] have confirmed the sequential process of exosomal miRNA transport as follows: (1) miRNA is selectively encapsulated within multivesicular bodies (MVBs); (2) MVBs fuse with the cell’s plasma membrane, releasing intraluminal vesicles (ILVs) into the extracellular environment, forming exosomes; (3) Exosomes merge with the plasma membrane of target cells, which subsequently internalize the exosomes through endocytosis; (4) Then exosomes specifically deliver miRNA to the 3′UTR end of target mRNA, consequently suppressing the expression of target genes (as illustrated in Figure 3).

### 4.2. Exosomal miRNA Function

The functions of exosomal miRNA can be broadly categorized into two groups. First, there is the conventional function [50], which involves regulating gene expression within cells through precise binding with target genes. The second category [51] is the ligand-like function, where miRNAs act as direct agonists of specific receptor families, interacting with proteins. MiRNAs enclosed within exosomes facilitate information transfer between cells, either through active cell secretion or the paracrine pathway. They exert direct or indirect influences on cellular functions, actively participating in the development and progression of diseases. In particular, they play a pivotal regulatory role in the context of CVDs [52]. This significance stems from both the inherent stability of exosomal miRNA and the protective effect offered by the exosome membrane. These factors contribute to the study of exosomal miRNA function and its potential applications in the diagnosis of CVDs.

## 5. Exosomal miRNAs from Different Sources and Myocardial Fibrosis

Emerging evidence suggests that most cardiac, vascular, and stem cells [53,54,55,56,57] release exosomes. Any alterations in the signaling molecules carried within exosomes or in the exosomal machinery can disrupt physiological homeostasis and ultimately lead to heart disease. Exosomes play a central role in many cardiac conditions, including myocardial infarction (MI), hypertrophic cardiomyopathy (HCM), and ischemia [58]. Recent studies indicate that the communication between cardiac cells via exosomes is disrupted during fibrosis, a key mediator of heart diseases [59]. These findings have spurred active research efforts aimed at comprehending the role of extracellular vesicles released by fibroblasts, as well as exosomes originating from various cell types, in contributing to cardiac dysfunction during fibrosis.

In this review, we provide a comprehensive summary of the roles of miRNAs in myocardial fibrosis, specifically focusing on the diverse roles of miRNAs found in exosomes derived from different cell types (as illustrated in Table 1).

### 5.1. Endothelial Progenitor Cells-Derived Exosomal miRNA

#### 5.1.1. miR-1246, miR-1290

Huang et al. [60] conducted a study in which they isolated exosomes from endothelial progenitor cells (EPCs) and observed a significant upregulation in the expression of EPC-derived exosomal miR-1246 and miR-1290. Mechanistically, miR-1246 and miR-1290 from EPC-derived exosomes were found to induce the upregulation of ELF5 and SP1 by targeting the promoter regions of their respective genes. This, in turn, promoted the transition of fibroblasts into endothelial cells and stimulated angiogenesis and proliferation of human cardiac fibroblasts (HCFs). The findings from this study suggest that miR-1246 and miR-1290 within EPC-derived exosomes enhance angiogenesis in the context of myocardial infarction. These improvements may facilitate the repair of post-myocardial infarction cardiac damage and the mitigation of cardiac fibrosis.

#### 5.1.2. miR-133

Lin et al. [61] previously discovered that exosomes derived from EPCs promote mesenchymal-endothelial transition (MEndoT) following hypoxia/reoxygenation (H/R) of EPCs. However, the underlying mechanism remained unclear. To address this, the researchers isolated EPCs from human peripheral blood and fibroblasts from rat hearts to investigate the relationship between EPC-derived exosomes, fibrosis, and MEndoT. The study revealed that H/R stimulation induces the upregulation of miR-133 within EPCs and their derived exosomes. This upregulated miR-133 is then incorporated into the exosomes with the assistance of YBX-1, and subsequently delivered to cardiac fibroblasts, promoting angiogenesis in cardiac fibroblasts and MEndoT.

#### 5.1.3. miR-218-5p, miR-363-3p

Numerous studies have demonstrated the potential of EPC-derived exosomes (EPC-Exos) in ameliorating myocardial fibrosis. In a study by Ke et al. [62], differentially expressed miRNAs between EPCs and exosomes were identified through miRNA-Seq data. The authors used quantitative real-time polymerase chain reaction (qRT-PCR) to confirm the differential expression of miRNAs identified in the miRNA-Seq data. The study revealed a significant increase in the expression of miR-363-3p and miR-218-5p in EPC-Exos following MI. MiR-218-5p and miR-363-3p were found to upregulate p53 expression by binding to the p53 promoter region and binding to the 3′UTR of junction-mediating and regulatory protein (JMY), leading to the downregulation of JMY expression, subsequently promoting mesenchymal-endothelial transition and inhibiting myocardial fibrosis.

### 5.2. Mesenchymal Stem Cell-Derived Exosomal miRNA

#### 5.2.1. miR-29c

Numerous lines of evidence suggest that mesenchymal stem cell (MSC) therapy holds promise as a potential treatment for DMD [91]. MSCs exert their therapeutic effects primarily through paracrine mechanisms, which involve the secretion of cytokines and extracellular vesicles [92]. Bier et al. [63] isolated mesenchymal stem cells derived from the placenta (PL-MSCs) and their secreted exosomes (PL-MSCs-Exos). They discovered that PL-MSCs-Exos promoted the differentiation of human myoblasts, suppressed the expression of fibrosis-related genes in myoblasts from DMD patients, reduced the expression of TGF-β in mdx mice, and alleviated fibrosis. These beneficial effects were attributed to the transfer of miR-29c from the exosomes.

#### 5.2.2. miR-22

Recent studies have highlighted the potential of MSCs to confer beneficial effects through paracrine mechanisms, whereby they release advantageous factors [93]. Exosomes play a pivotal role in the transport of these paracrine factors, and the miRNAs encapsulated within exosomes can orchestrate various biological processes in recipient cells [42]. In a particular study by Feng [64], it was observed that MSCs, after ischemic preconditioning (IPC), release exosomes enriched with miR-22. Subsequently, when these exosomes are internalized by cardiomyocytes, miR-22 exerts its function by directly targeting methyl CpG binding protein 2 (Mecp2), thereby inhibiting cardiomyocyte apoptosis. In vivo experiments further validated the capacity of MSC-derived exosomes (MSC-Exos) to significantly mitigate cardiac fibrosis.

#### 5.2.3. miR-126

Recent studies have demonstrated that adipose-derived stem cells (ADSCs) possess the capacity to secrete numerous cytokines and growth factors, which contribute to their reparative potential following stem cell mobilization or transplantation—a valuable approach for treating MI [94]. However, when ADSCs are transplanted into the inflammatory and ischemic microenvironment of acute myocardial infarction (AMI), the outcomes have often been suboptimal [95,96]. In a study by Luo [65], it was observed that overexpressing miR-126 in ADSC-derived exosomes had a beneficial impact on hypoxia-induced cardiomyocyte injury. This effect was achieved by not only downregulating the expression of inflammatory factors in cardiomyocytes but also inhibiting the expression of fibrosis-related proteins in H9c2 cells. Furthermore, it significantly facilitated microvascular generation and migration.

#### 5.2.4. miR-146a

Several studies have demonstrated that early growth response factor 1 (EGR1) can trigger myocardial damage following AMI. Through bioinformatics analysis, it has been revealed that miR-146a has the potential to regulate the expression of EGR1. In a study conducted by Pan et al. [66], exosomes derived from ADSCs were isolated. These exosomes, enriched with miR-146a, exhibited a significant capacity to suppress AMI-induced apoptosis, inflammation, and fibrosis. MiR-146a exerts its effects by interacting with the 3′-UTR of EGR1, leading to the downregulation of post-transcriptional EGR1 expression and ultimately mitigating AMI or hypoxia-induced TLR4/NFκB signaling.

#### 5.2.5. miR-210

Hypoxic preconditioning has been shown to enhance the biological activity of MSCs, thus improving the effectiveness of MSC transplantation in treating MI [97,98,99]. In a past study [67], the researchers explored whether exosomes derived from hypoxia-treated MSCs (Exo^H^) outperformed those from normal-oxygen-treated MSCs (Exo^N^) in terms of myocardial repair. The results indicated that Exo^H^ significantly increased the survival rate of mice with MI, reduced infarct size, and improved cardiac function. Following microarray-based miRNA profiling of Exo^H^ and Exo^N^ and reference to the published literature, the study focused on miR-21, which is highly upregulated under hypoxic conditions. According to literature reports, neutral sphingomyelinase 2 (nSMase2) serves as a critical regulator of exosome biogenesis and secretion [100]. To ascertain whether hypoxia-enhanced exosomal miR-210 expression is modulated in an nSMase2-dependent manner, the authors inhibited nSMase2 activity in MSCs under hypoxic conditions using the selective inhibitor GW4869. Subsequently, they observed a dose-dependent reduction in miR-210 levels in exosomes following treatment with GW4869. Notably, the expression of neutral sphingomyelinase 2 (nSMase2) in Exo^H^ was significantly elevated, leading to an increase in the expression of miR-210. This upregulation of miR-210 resulted in heightened vascular density, reduced cardiomyocyte apoptosis, decreased fibrosis, and enhanced recruitment of cardiac progenitor cells in the infarcted heart. The authors postulate that the enhanced cardioprotective effect of exosomes derived from hypoxia-treated MSCs can be attributed to the increased expression of miR-210.

#### 5.2.6. miR-24

Shao [68] found that miR-24 is a major component of umbilical cord mesenchymal stem cell (UMSC)-derived exosomes by miRNA sequencing, and a bioinformatics analysis suggested that Bim may be a target gene for miR-24. Their results suggest that UMSC inhibits cardiac fibrosis and restores cardiac function through the exosome/miR-24/Bim pathway.

#### 5.2.7. miR-671

miR-671 [69] contained in exosomes derived from ADSCs inhibited Smad2 phosphorylation by targeting the transformation of growth factor β receptor 2 (TGFBR2), improved cardiomyocyte viability, reduced cardiomyocyte apoptosis, and alleviated myocardial fibrosis and inflammation.

### 5.3. Macrophages-Derived Exosomal miRNA

#### miR-155

Inflammation plays a crucial role in heart damage. Wang et al. [70] observed an increase in miR-155 expression within cardiac macrophages and fibroblasts in mice following MI. Notably, the primary miR-155 transcript (pri-miR-155) was exclusively expressed in macrophages. Macrophage-derived exosomes facilitate the transfer of miR-155 to cardiac fibroblasts. Once in fibroblasts, miR-155 binds to the 3′UTR terminus of Son of Sevenless 1 (Sos1), a dual guanine nucleotide exchange factor crucial for cell growth regulation and interaction with Ras [101]. This interaction enhances ERK activity by increasing the formation of the Grb2-Sos1 complex [102], consequently promoting cell proliferation. However, miR-155 downregulates the level of Sos1 protein in cardiac fibroblasts, thereby inhibiting the proliferation of cardiac fibroblasts. Simultaneously, it promotes inflammation by reducing the expression of Suppressor of Cytokine Signaling 1.

### 5.4. Endothelial Cells-Derived Exosomal miRNA

#### 5.4.1. miR-19a-3p

Low-energy shock wave therapy (SWT) is a highly effective non-invasive method, well-documented for its efficacy in inducing fibroblast angiogenesis [103] and its associated benefits. While there is a wealth of literature on the positive effects of SWT, there remains limited understanding of the underlying mechanisms at play. Recently, a study [104] proposed that RNA release via exosomes may underpin the actions of SWT. Another article [71] discovered that mechanical stimulation of ischemic muscle with SWT triggers the release of extracellular vesicles by endothelial cells. Upon characterization of these vesicles, the researchers identified exosomes with angiogenic properties, promoting endothelial angiogenesis and cell proliferation by activating protein kinase B (Akt) and extracellular-signal regulated kinase (ERK).

Through miRNA and transcriptomic sequencing of these exosomes, the authors pinpointed miR-19-3a as the key molecule within them. Injecting miR-19-3a into the myocardium of mice undergoing left anterior descending (LAD) artery ligation induced angiogenesis, inhibited fibrosis, and improved cardiac function following ischemia.

#### 5.4.2. miR-10b-5p

Current approaches to treating cardiac fibrosis mainly revolve around cell-based therapies. In a recent study [72], researchers explored changes in the antifibrotic effects of ECFC-derived exosomes under hypoxic conditions and the underlying mechanisms. They collected ECFC-derived exosomes under normal oxygen conditions (nor-exo) and hypoxia (hyp-exo) regulation. Their findings revealed a significant reduction in the content of miR-10b-5p in hyp-exo compared to nor-exo. Moreover, the expression of N-SMase2 was also markedly decreased. A dual-luciferase reporter gene assay confirmed that fibrosis-related genes, SMAD-specific E3 ubiquitin protein ligase 1 (Smurf1) and histone deacetylase 4 (HDAC4), were downstream targets of miR-10b-5p. This suggests that hypoxia inhibited the expression of miR-10b-5p, consequently diminishing its anti-fibrotic effects.

### 5.5. Cardiomyocytes-Derived Exosomal miRNA

#### 5.5.1. miR-208a

Recent studies have revealed that the pathogenesis of cardiac fibrosis is not solely associated with activated cardiac fibroblasts [70], as cardiomyocytes may also play a crucial role [105]. Nevertheless, the specific role and mechanisms of cardiomyocytes in cardiac fibrosis following myocardial infarction remain unclear.

In a study by Yang [73], a key gene, miR-208a, was identified through a comparison of differentially expressed cardiac-specific miRNAs in Doxorubicin-induced fibrotic rat models and LAD coronary ligation-induced fibrotic rat models. The researchers then transferred exosomes isolated from cardiomyocytes in the fibrosis model into rats, where they observed an upregulation of miR-208a in both cardiomyocytes and cardiomyocyte-derived exosomes in the two cardiac fibrosis models. Notably, these exosomes can be transferred to cardiac fibroblasts, binding to the target gene, dual-specificity tyrosine-phosphorylation-regulated kinase 2 (Dyrk2), thereby promoting fibroblast proliferation and myofibroblast differentiation, ultimately contributing to cardiac fibrosis.

#### 5.5.2. miR-494-3p

Recent research [73] has highlighted the role of cardiomyocyte-fibroblast cross-talk in MI-induced heart failure. Cardiomyocytes have been identified as key contributors to cardiac fibrosis through the secretion of exosomes containing specific miRNAs. Previous studies [106] have established that miRNAs from cardiomyocytes regulate Pellino1 (Peli1)-mediated NF-κB and AP-1 activation. In an extension of this work, Tang et al. [74] discovered that the absence of Peli1 in cardiomyocyte-derived exosomes mitigates pressure overload and mechanical stretch-induced cardiac fibrosis. Through miRNA microarray analysis and qPCR of exosomes released by cardiomyocytes, the researchers identified miR-494-3p as a downstream target of Peli1. Mechanistically, Peli1 facilitated the expression of miR-494-3p via NF-κB/AP-1 in cardiomyocytes. Subsequently, miR-494-3p induced activation in cardiac fibroblasts by inhibiting PTEN and enhancing the phosphorylation of AKT, SMAD2/3, and ERK, thus promoting fibrosis.

#### 5.5.3. miR-378

Another study investigated the impact of exosomes released by cardiomyocytes on fibroblasts in a model of fibrosis induced by transverse aortic constriction (TAC) and stretching silicon dishes [75]. The research revealed that introducing miR-378 mimics (Agomir) into cardiomyocytes and co-culturing cardiomyocytes with fibroblasts effectively suppressed fibrotic responses in both models. These responses included mitigating myocardial fibrosis, reducing fibroblast proliferation, and decreasing the expression of collagen and matrix metalloproteinases (MMPs) at the protein and gene levels. The study unveiled that miR-378 derived from cardiomyocytes is conveyed in extracellular vesicles, thereby inhibiting p38 (mitogen-activated protein kinase) MAPK phosphorylation in fibroblasts through the targeting of MKK6. This process contributes to the attenuation of myocardial fibrosis.

#### 5.5.4. miR-29b, miR-455

Exercise has been demonstrated to alleviate diabetes complications, including heart dysfunction in individuals with diabetes [107]. Studies have shown that the deletion of the matrix metalloproteinase-9 (MMP9) gene can enhance the contractile function [108] of cardiomyocytes in diabetic patients, thereby promoting cardiac repair processes [109]. Consequently, the molecular mechanism underlying the positive effects of exercise may be associated with the reduction of MMP9 levels in the aorta [110].

Exosomes are known to play a pivotal role in heart repair and are considered promising therapeutic candidates. As a result, some researchers [76] argue that the molecular mechanism behind the benefits of exercise could be linked to the release of exosomes containing miRNAs during physical activity. These miRNAs are believed to inhibit the expression of MMP9. In a study by Chaturvedi et al. [76], it was found that miR-455 and miR-29b present in exosomes secreted by cardiomyocytes could bind to the 3′UTR of MMP9, leading to the downregulation of MMP9 expression. This downregulation helps alleviate the detrimental effects of MMP9 on extracellular matrix remodeling and reduces fibrotic responses.

#### 5.5.5. miR-210-3p

In a study by Hao et al. [77], miRNA sequencing analysis revealed a significant increase in the expression of miR-210-3p in exosomes secreted by atrial myocytes and the serum of patients with AF. Knocking down miR-210-3p was found to reduce the incidence of AF, inhibit the proliferation of atrial fibroblasts, and reduce collagen synthesis, thus improving Ang II-induced atrial fibrosis. Further analysis through mRNA sequencing and dual-luciferase reporter gene assays identified glycerol-3-phosphate dehydrogenase-like (GPD1L) as the downstream target of miR-210-3p. GPD1L was shown to regulate atrial fibrosis through the phosphoinositide-3-kinase (PI3K)/AKT signaling pathway.

#### 5.5.6. miR-23a

Exosomes derived from atrial myocytes of Ang II-treated mice with overexpressed NRON were isolated and co-cultured with RAW264.7 macrophages. Subsequently, mouse fibroblasts were cultured using the medium conditioned by RAW264.7 macrophages treated as described above. In a study conducted by Li [78], it was observed that NRON reduced the levels of miR-23a in exosomes derived from atrial myocytes by inhibiting NFATc3 nuclear transport. This led to the polarization of M2 macrophages, downregulation of fibrosis markers in atrial fibroblasts, and the mitigation of atrial fibrosis.

### 5.6. Cardiosphere-Derived Cells-Derived Exosomal miRNA

#### miR-92a

After heart injury, Cardiosphere-derived cells (CDCs) are known to initiate the repair process aimed at mending the damaged heart [111,112]. Clinical studies have demonstrated that CDCs exert their therapeutic effects by releasing extracellular vesicles, including exosomes, which stimulate anti-inflammatory, anti-fibrotic, pro-angiogenic, and pro-cardiogenic pathways [113]. This has positioned CDCs as potential candidates for enhancing biological activity and treating various diseases. However, the role of CDCs can be inconsistent, and the underlying molecular mechanisms remain unclear, posing challenges to their clinical applications. In a study by Ibrahim [79], transcriptomic analysis of CDCs obtained from human donors revealed a correlation between the therapeutic efficacy of CDCs and the Wnt/β-catenin signaling pathway, along with the levels of β-catenin protein. Additionally, significantly increased expression of miR-92a, a known enhancer of the Wnt/β-catenin pathway, was observed in exosomes derived from CDCs. Transplantation of skin fibroblasts that overexpress β-catenin and the transcription factor Gata4 into mdx mouse models of DMD resulted in improvements in skeletal-muscle fibrosis.

### 5.7. Cardiac Progenitor Cells-Derived Exosomal miRNA

#### 5.7.1. miR-133a

Transplantation of mature cells or stem cells is a widely adopted approach for the treatment of cardiovascular diseases [114]. Among these, the use of cardiac stem/progenitor cells (CSCs/CPCs) with specific surface markers and substantial differentiation potential holds promise [115]. MiR-133a [80] is a muscle-specific miRNA whose expression increases progressively during in vitro cardiac differentiation of adult cardiac progenitor cells (CPCs). Overexpression of miR-133a confers protection upon CPCs by targeting the pro-apoptotic genes Bim and Bmf, preventing cell death. In a rat model of myocardial infarction, the secretion of miR-133a by CPCs (miR-133a-CPCs) significantly enhances cardiac function by reducing fibrosis and hypertrophy, promoting angiogenesis, and increasing cardiomyocyte proliferation. Notably, these beneficial effects of miR-133a-CPCs are attributed to the transportation of miR-133a within exosomes.

#### 5.7.2. miR-146a-5p

The exosomes released by cardiac resident mesenchymal progenitor cells (CPCs) are enriched with miR-146a-5p, which plays a vital role in promoting cardiac repair following AMI and ischemia-reperfusion injury [116]. In contrast, the combined administration of Dox and Trz induced myocardial fibrosis, CD68+ inflammatory cell infiltration, increased inducible nitric oxide synthase expression, and led to left ventricular dysfunction in rats. However, treatment with isolated CPCs [81] and purified exosomes obtained from patients undergoing cardiac surgery successfully reversed these adverse changes. This reversal can be attributed to the abundance of miR-146a-5p in exosomes, which suppresses the expression of genes encoding key signaling mediators within the inflammatory and cell-death pathways, including Traf6, Smad4, Irak1, Nox4, and Mpo; promotes the survival of cardiomyocytes; and mitigates cardiotoxicity.

### 5.8. Pluripotent Stem Cell-Derived Exosomal miRNA

#### 5.8.1. miR-290-295 Cluster

Pluripotent stem cells with unparalleled differentiation potential include embryonic stem cells (ESCs) and induced pluripotent stem cells (iPSCs), offering substantial promise for cardiac regeneration [117]. Currently, exogenous stem cell therapy is a prominent approach for treating cardiovascular diseases.

Mouse embryonic stem cell-derived exosomes (mESC Ex) transport miR-294 [55] from ESCs to CPCs, thereby enhancing the survival and proliferation of CPCs. These exosomes also promote angiogenesis and cardiomyocyte survival, reduce post-infarction fibrosis, and mitigate the risks associated with the direct transplantation of embryonic stem cells or their derivatives, such as teratoma formation.

#### 5.8.2. miR-373

IPSCs possess the capacity to differentiate into various progenitor cell types, paving the way for the advancement of cell therapies. Cardiac progenitors derived from iPSCs have been utilized in both MI animal models [118] and human subjects [119], showing promising outcomes through the secretion of EVs containing multiple miRNAs, proteins, and other factors that mediate cardiac protection. In this study [82], the authors generated pluripotent CPCs from human-induced pluripotent stem cells (hiPSCs) using the small molecule ISX-9, known for its antioxidant and regenerative properties. It was observed that ISX-9-induced CPCs secreted EVs (160–170 nm in diameter) enriched with exosome-specific markers such as Tsg101, CD9, Hsp70, flotillin-1, and calnexin (referred to as EV-CPC^ISX-9^). Furthermore, miR-373, highly abundant in EV-CPCISX-9, was found to target two genes, growth differentiation factor 11 (GDF-11) and Rho Associated Coiled-Coil Containing Protein Kinase 2 (ROCK-2), thereby inhibiting the expression of fibrosis-related genes (MMP-2, TIMP-2, TIMP-1, FN1, CTGF, and MMP-9), suppressing fibroblast-to-myofibroblast transdifferentiation, and exerting significant anti-fibrotic effects.

### 5.9. Immune Cells-Derived Exosomal miRNA

#### miR-142-3p

Sustained inflammation mediated by the infiltration of CD4+ T cells into the heart is strongly associated with the progression of cardiac fibrosis and dysfunction [120]. Studies have also indicated that the deactivation of CD4+ T cells can mitigate cardiac hypertrophy and fibrosis induced by pressure overload [121]. These findings suggest that activated CD4+ T cells contribute to adverse cardiac remodeling, but the specific mediators of this process require further investigation.

miR-142-3p, enriched in exosomes (referred to as CD4-activated exosomes) from activated CD4+ T cells, directly targets and inhibits the expression of adenomatous polyposis coli (APC). APC is a known negative regulator of the WNT signaling pathway. By inhibiting APC, miR-142-3p activates the WNT signaling pathway and subsequently stimulates myoblasts [83]. This activation, however, can induce the transformation of myocardial fibroblasts into myofibroblasts, exacerbating myocardial fibrosis and contributing to post-infarction dysfunction.

### 5.10. Serum-Derived Exosomal miRNA

#### 5.10.1. miR-21

Human peripheral blood-derived exosomes are particularly promising for use as a drug delivery system due to their ability to carry miRNAs, ease of obtainment, and applicability in this context. One miRNA of significant interest is miRNA-21, which plays a crucial role in the development of cardiac fibrosis in response to MI. It achieves this by targeting various molecules, including SMAD family member 7 (Smad7), sprout RTK signaling antagonist 1 (SPRY1), and phosphatase and tensin homolog (PTEN) [122,123,124]. MiRNA-21 has also been demonstrated to enhance fibrosis in a mouse MI model [84], making it an ideal candidate for therapeutic applications due to its role in regulating gene expression during fibrosis.

#### 5.10.2. miR-320a

In Wang’s study [85], serum exosomes from 10 patients with CHF and 5 healthy individuals were analyzed. The results revealed that CHF patients exhibited higher levels of serum miR-320a expression and soluble growth stimulation expressed gene 2 (sST2) in comparison to the healthy controls. Notably, the levels of serum miR-320a were found to be correlated with clinical markers of CHF. Furthermore, miR-320a expression in exosomes was also notably elevated in CHF patients when compared to healthy individuals. In HEH2 cells, the overexpression of serum exosomal miR-320a was observed to promote the proliferation of myocardial fibroblasts by upregulating the expression of collagen I, collagen III, α-smooth muscle actin (α-SMA), and phosphorylated (p)-mTOR (ser 2448)/mTOR, p-Akt (ser 473)/Akt, p-Akt (thr 308)/Akt, as well as the phosphoinositide-3-kinase catalytic α polypeptide gene (PIK3CA), thus activating the PIK3CA/Akt/mTOR signaling pathway. The findings suggest that miR-320a in serum exosomes may hold promise as a potential biomarker for CHF.

#### 5.10.3. miR-425, miR-744

In a recent investigation [86], researchers investigated the expression of nine candidate miRNAs within plasma exosomes from heart failure patients. Their observations revealed distinct alterations in the levels of miR-21, miR-425, and miR-744. Moreover, the study revealed that the downregulation of miR-425 and miR-744, which are associated with elevated expression of collagen 1 and α-SMA, also manifested in Ang II-stimulated cardiac fibroblasts. This implies that miR-425 and miR-744 may serve pivotal roles in the processes of cardiac fibrosis and heart failure. They do so by modulating the expression of TGFβ1, consequently influencing collagen production and the overall fibrotic response.

#### 5.10.4. miR-124-3p

In a study by Zhu [87], miR-124-3p with functional significance was discovered in exosomes extracted from the plasma of three patients with atrial fibrillation and three patients with sinus rhythm (SR). This miRNA directly targets AXIN1, leading to a reduction in its expression. As a result, miR-124-3p upregulates the expression of collagen I and α-SMA by modulating the WNT/β-catenin signaling pathway, enhancing the viability and proliferation of myocardial fibroblasts and exacerbating myocardial fibrosis.

### 5.11. Adipocyte-Derived Exosomal miRNA

#### miR-23a-3p

Ang II binds to its heart-specific receptor, Ang II receptor type 1 (AT1R), stimulating the differentiation of fibroblasts into myofibroblasts and promoting collagen secretion by cardiac fibroblasts, thus contributing to the progression of myocardial fibrosis [125,126]. Adipose tissue plays a significant role in the development and progression of cardiovascular diseases by producing various hormones and cytokines that regulate the function of distant organs [127,128]. Specifically, epididymal white adipose tissue (eWAT) releases pro-fibrotic cytokines and adipokines that can accelerate heart aging [129].

Under the influence of Ang II, exosomes derived from adipocytes transport miR-23a-3p to CFs and target RAP1, which promotes excessive collagen deposition and the transformation of fibroblasts into myofibroblasts. These processes actively contribute to the pathogenesis of myocardial fibrosis and cardiac dysfunction [88].

### 5.12. Others

#### 5.12.1. miR-29b

After implanting a biocompatible microneedle (MN) patch loaded with exosomes containing miR-29b mimics with anti-fibrotic activity into a mouse MI model [89], the inflammation in the infarcted heart reduced, the infarct size diminished, the expression of fibrosis-related proteins decreased, fibrosis was inhibited, and cardiac function improved.

#### 5.12.2. miR-450a-2-3p

Exosomes extracted from pericardial fluid (PF) surrounding the heart are enriched with LINC00636 and miR-450a-2-3p [90], both of which are involved in the anti-fibrotic process. Overexpression of miR-450a-2-3p inhibits the expression of MAPK1 in cardiac fibroblasts, thereby inhibiting the expression of α-SMA, collagen I, and collagen III, preventing the proliferation of cardiac fibroblasts and alleviating myocardial fibrosis.

## 6. Conclusions and Prospect

Myocardial fibrosis is closely associated with high cardiovascular mortality and a low survival rate. It serves as a hallmark of heart remodeling and represents a critical pathological basis for the development of heart diseases and the deterioration of heart function. Effectively managing myocardial fibrosis is of paramount importance for preventing and treating heart diseases and enhancing patients’ quality of life. Exosomes, as emerging mediators of intercellular communication, play a pivotal role in the initiation and progression of myocardial fibrosis by delivering miRNAs and regulating protein expression in target cells. Although there is a relatively low abundance of miRNA in EVs, there is approximately one miRNA per 10–100 EVs [130,131], its functions of regulatory [42], being biomarkers [132], and delivering drugs [133] have been well documented. Given the significant influence of miRNAs on both adaptive and maladaptive heart remodeling and the potential of exosomes as carriers for these miRNAs, exosomal miRNAs hold great promise as a burgeoning research avenue in the field of myocardial fibrosis treatment and cardiovascular medicine as a whole, thus offering a promising novel approach for the diagnosis and treatment of cardiovascular diseases.

Nevertheless, in the treatment of complex CVDs, the versatile biological functions of exosomal miRNAs introduce an element of therapeutic variability. Exosomes, as carriers for targeted biological therapies, offer the advantage of multi-directional target regulation and hold significant promise. Conversely, several challenges related to precise expression and targeted delivery must still be addressed. Furthermore, exosomes carry a multitude of variable miRNAs that regulate intricate signal transduction pathways, exerting a global regulatory influence on recipient cells. Yet, due to the complexity of this regulation, fully comprehending the functions of exosomal miRNAs remains a challenge.

Currently, various methods for exosome isolation exist, but it is important to recognize that different purification strategies may influence the detection of exosomal miRNA. Therefore, future investigations should explore the measurement of miRNA-enriched exosome subsets and conduct quantitative studies to determine functional thresholds for exosome miRNAs. A deeper comprehension of the biological functions of exosomal miRNAs and their regulatory mechanisms in myocardial fibrosis could lay the foundation for their clinical translation.

## Figures and Tables

**Table 1:** Regulatory-different cell-derived miRNAs associated with cardiac fibrosis.

The Origin of Exosomes	miRNA	Target Gene/Pathway	Role	Disease Models	References
Endothelial progenitor cells	miR-1246, miR-1290	ELF5 and SP1	Anti-fibrosis	Myocardial infarction	[60]
	miR-133	YBX-1	Anti-fibrosis	Hypoxia/reoxygenation	[61]
	miR-218-5p, miR-363-3p	p53 and JMY	Anti-fibrosis	Myocardial infarction	[62]
Mesenchymal stromal cells	miR-29c	TGF-β	Anti-fibrosis	Duchenne muscular dystrophy	[63]
	miR-22	Mecp2	Anti-fibrosis	Ischemic heart disease	[64]
	miR-126	Unknown	Anti-fibrosis	Acute myocardial infarction	[65]
	miR-146a	EGR1/TLR4/NFκB	Anti-fibrosis	Acute myocardial infarction	[66]
	miR-210	HIF-1α	Anti-fibrosis	Myocardial infarction	[67]
	miR-24	Bim	Anti-fibrosis	Myocardial infarction	[68]
	miR-671	TGFBR2	Anti-fibrosis	Myocardial infarction	[69]
Macrophages	miR-155	FoxO3a	Pro-fibrosis	Uremic cardiomyopathy	[70]
Endothelial cells	miR-19a-3p	Akt/ERK	Anti-fibrosis	Myocardial infarction	[71]
	miR-10b-5p	Smurf1 and HDAC4	Anti-fibrosis	Ischemic heart disease	[72]
Cardiomyocytes	miR-208a	Dyrk2	Pro-fibrosis	Fibrosis	[73]
	miR-494-3p	PTEN	Pro-fibrosis	Fibrosis	[74]
	miR-378	MKK6/P38 MAPK pathway	Anti-fibrosis	Fibrosis	[75]
	miR-29b, miR-455	MMP9	Anti-fibrosis	Type 2 diabete	[76]
	miR-210-3p	GPD1L/PI3K/AKT signaling pathway	Pro-fibrosis	Atrial fibrillation	[77]
	miR-23a	Unknown	Pro-fibrosis	Atrial fibrillation	[78]
Cardiosphere-derived cells	miR-92a	BMP2	Anti-fibrosis	Duchenne muscular dystrophy	[79]
Cardiac progenitor cells	miR-133a	Bim, Bmf, bFgf and Vegf	Anti-fibrosis	Myocardial infarction	[80]
	miR-146a-5p	Traf6, Smad4, Irak1, Nox4, and Mpo	Anti-fibrosis	Doxorubicin/trastuzumab-induced cardiac toxicity	[81]
Pluripotent stem cells	miR-290-295 cluster	Unknown	Anti-fibrosis	acute myocardial infarction	[55]
	miR-373	GDF-11 and ROCK-2	Anti-fibrosis	Myocardial infarction	[82]
Immune cells	miR-142-3p	APC/WNT	Pro-fibrosis	Myocardial infarction	[83]
Serum	miR-21	Smad7, PTEN and MMP2	Pro-fibrosis	Myocardial infarction	[84]
	miR-320a	PIK3CA/Akt/mTOR signaling pathway	Pro-fibrosis	Chronic heart failure	[85]
	miR-425, miR-744	TGF-β1	Anti-fibrosis	Heart failure	[86]
	miR-124-3p	AXIN1/WNT/β-catenin signaling pathway	Pro-fibrosis	Atrial fibrillation	[87]
Adipocytes	miR-23a-3p	RAP1	Pro-fibrosis	Fibrosis	[88]
Others	miR-29b	Unknown	Anti-fibrosis	Myocardial infarction	[89]
	miR-450a-2-3p	MAPK1	Anti-fibrosis	Atrial Fibrillation	[90]
